# Mitochondrial mutations in maternally inherited hearing loss

**DOI:** 10.1186/s12881-017-0389-4

**Published:** 2017-03-20

**Authors:** Hideki Mutai, Takahisa Watabe, Kenjiro Kosaki, Kaoru Ogawa, Tatsuo Matsunaga

**Affiliations:** 1 Division of Hearing and Balance Research, National Institute of Sensory Organs, National Hospital Organization Tokyo Medical Center, 2-5-1 Higashigaoka, Meguro, Tokyo, 152-8902 Japan; 2 Department of Otolaryngology, Head and Neck Surgery, Keio University School of Medicine, 35 Shinanomachi, Shinjuku, Tokyo, 160-8582 Japan; 3 Center for Medical Genetics, Keio University School of Medicine, 35 Shinanomachi, Shinjuku, Tokyo, 160-8582 Japan

**Keywords:** Mitochondrial deafness, Maternal inheritance, *MTTS1*

## Abstract

**Background:**

Although the mitochondrial DNA (mtDNA) mutations m.1555A > G and m.3243A > G are the primary causes of maternally inherited sensorineural hearing loss (SNHL), several other mtDNA mutations are also reported to be associated with SNHL.

**Methods:**

Screening of m.1555A > G and m.3243A > G mutations was performed for 145 probands. Nine probands fulfilled the following criteria: 1) bilateral and symmetric SNHL, 2) ≥ 4 family members with SNHL with a maternal trait of inheritance in ≥ 2 generations, 3) onset of SNHL before the age of 40 years, 4) high-frequency SNHL, and 5) no record of environmental factors related to SNHL. Sequencing of additional mtDNA regions was performed for five subjects meeting the clinical criteria, but the screening results were negative.

**Results:**

Among the nine cases meeting the five clinical criteria detailed above, three had the m.1555A > G mutation in *MTRNR1*, one had a m.3243A > G mutation in *MTTL1*, and one case had a m.7511T > C mutation in *MTTS1*. In the family with the m.7511T > C mutation, penetrance of SNHL among maternally related subjects was 9/17 (53%). The age at onset varied from birth (congenital) to adulthood. Hearing levels varied from normal to moderately impaired, unlike previously reported subjects with this mutation, where some maternal family members presented with profound SNHL. Family members with the m.7511T > C mutation and SNHL did not exhibit any specific clinical characteristics distinct from those of other individuals with SNHL and different mtDNA mutations. Among the 136 probands who did not meet the criteria detailed above, one case had the m.1555A > G mutation, and three cases had the m.3243A > G mutation.

**Conclusions:**

Since five of nine probands with the clinical criteria used in this study had mtDNA mutations, these criteria may be helpful for identification of candidate patients likely to have mtDNA mutations.

**Electronic supplementary material:**

The online version of this article (doi:10.1186/s12881-017-0389-4) contains supplementary material, which is available to authorized users.

## Background

Hearing loss is one of the most prevalent sensory disorders. Genetic factors are thought to account for more than half of congenital and childhood-onset hearing loss [1, 2], and mutations of mitochondrial DNA (mtDNA) are associated with maternally inherited sensorineural hearing loss (SNHL). Human mtDNA is a double-stranded, circular molecule, encoding 13 protein subunits, two ribosomal RNAs (rRNAs), and 22 transfer RNAs (tRNAs) [3]. Among the mutations in mitochondrial genes, m.1555A > G in *MTRNR1* (also known as *12SrRNA*), and m.3243A > G in *MTTL1* (*tRNA* ^*Leu(UUR)*^) are relatively frequent causes of SNHL [4]. The m.1555A > G mutation in *MTRNR1* is associated with aminoglycoside ototoxicity and nonsyndromic SNHL, while *MTTL1* m.3243A > G is associated with mitochondrial encephalomyopathy, lactic acidosis, and stroke-like episodes (MELAS); maternally inherited diabetes and deafness syndrome (MIDD); and chronic progressive external ophthalmoplegia (CPEO). In addition, several other mitochondrial mutations, including m.1494C > T in *MTRNR1*, and m.7445A> G, 7472insC, and 7511T > C in *MTTS1* (*tRNA* ^*Ser(UCN)*^) have been associated with nonsyndromic SNHL [4]. Furthermore, m.8344A> G and m.8356T > C mutations in *MTTK* (*tRNA* ^*Lys*^) [5]*,* the m.14709T > C mutation in *MTTE* (*tRNA* ^*Glu*^) [6], and several variants in *MTTH* (*tRNA* ^*His*^) [7] and *MTTS2* (*tRNA* ^*Ser(AGY)*^) [8] have also been confirmed, or are reported, as associated with nonsyndromic or syndromic SNHL.

The clinical features of mitochondrial hearing loss are: 1) maternal inheritance; 2) hearing loss is always sensorineural and primarily symmetrical, with involvement of the higher frequencies, or all frequencies; 3) variable penetrance and severity, even within families; and 4) in general, childhood onset (postlingually) [4].

In this study, we performed mtDNA mutation analysis on two rRNA and 22 tRNA genes, including *MTRNR1, MTTL1, MTTS1, MTTK, MTTH, MTTS2,* and *MTTE*, variants in which have previously been reported to be associated with SNHL. We also investigated the clinical characteristics of family members with the m.7511A > C mutation. We propose criteria to identify candidate patients likely to have mitochondrial mutations.

## Methods

### Subjects

The present study included 171 consecutive subjects from 145 families who visited the Department of Otolaryngology at Keio University Hospital with complaints of hearing loss and were then referred to the Center for Medical Genetics for genetic evaluation. Based on the clinical features of mitochondrial deafness (as reviewed by Kokotas et al. [4]), patients were selected for comprehensive mtDNA analysis if they met the following clinical criteria: 1) bilateral and symmetric SNHL; 2) at least four family members with SNHL with a maternal trait of inheritance for at least two generations; 3) onset of SNHL before the age of 40 years; 4) high-frequency SNHL (≥15 dB difference in hearing levels between the means of 0.5 and 1 kHz and the means of 4 and 8 kHz, assessed by pure-tone audiometry. Both “gently sloping” and “steeply sloping” audiograms were taken into account); and 5) no record of environmental factors related to hearing loss, such as infectious diseases, premature birth, or newborn meningitis. The audiometric configuration of criterion 4) and severity of hearing loss were determined according to the guidelines of the GENEDEAF study group (http://hereditaryhearingloss.org). Patients with a history of use of ototoxic drugs were included in this study, because some drugs are associated with mitochondrial SNHL. Kaplan-Meier curves were plotted using R platform.

### Genetic analysis

Genomic DNA was extracted from blood samples using the Gentra Puregene Blood kit (QIAGEN, Hamburg, Germany). Detection of the m.1555A > G and m.3243A > G mutations in all 145 probands was performed by restriction fragment length polymorphism (RFLP) analysis, as previously described [9, 10]. Samples from five subjects, who met the clinical criteria and were not found to have mtDNA mutations by RFLP, were subjected to mutation analysis of mtDNA regions, including two rRNA and 22 tRNA genes by nested PCR, as described in [11] with modification. The nested-PCR was performed using PrimeSTAR HS DNA polymerase (Takara Bio, Shiga, Japan) and the following program: 98 °C for 10 min; 37 cycles of 98 °C for 10 s, 62 °C for 10 s, and 72 °C for 1 min; and then 72 °C for 3 min. PCR primers are listed in Additional file 1. Amplicons were sequenced using an ABI 3730 DNA sequence analyzer, using the ABI Prism Big Dye Terminator Cycle Sequencing kit (Applied Biosystems, MA, USA). Sequences were characterized using SeqScape software v2.6 (Applied Biosystems) and DNASIS Pro (Hitachisoft, Tokyo, Japan) with rCRS NC_012920.1.

## Results

### mtDNA analysis

**Fig. 1:**
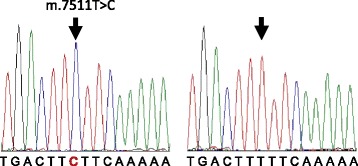
Electropherograms of part of the *MTTS1* sequence. Sequence from the proband (III-5, left; showing the m.7511T > C mutation) and a control subject with normal hearing (*right*) are shown

Among 145 probands who visited the Department of Otolaryngology at Keio University Hospital, nine fulfilled the five clinical criteria detailed in the Methods section and were, therefore, analyzed for mtDNA mutations. Among these nine individuals, homoplasmic m.1555A > G and heteroplasmic m.3243A > G mutations were identified in three cases and a single case, respectively by RFLP analysis. Samples from the remaining five cases, who met the clinical criteria but did not have m.1555A > G or m.3243A > G mutations, were subjected to Sanger sequencing analysis of mtDNA regions, including two rRNA and 22 tRNA genes, as described in the Methods section. One case had a homoplasmic (or a high level heteroplasmic) m.7511T > C mutation in *MTTS1* (Fig. 1). Other mtDNA variants detected in the five subjects are presented in Additional file 2. No novel or possible pathological mutations, nor any unknown variants, were detected in the remaining four subjects.

RFLP screening revealed homoplasmic m.1555A > G and heteroplasmic m.3243A > G mutations in one and three patients, respectively, among 136 probands who failed to meet the five criteria. The clinical features of probands with the m.1555A > G and m.3243A > G mutations are presented in Additional file 3.

### Clinical findings in the family with the m.7511T > C mutation

**Fig. 2:**
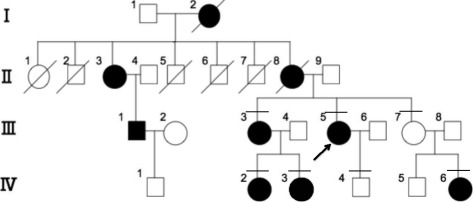
Pedigree of the family with the m.7511T > C mutation. *Arrow* indicates the proband. Subjects with horizontal bars above the symbols underwent genetic and clinical analyses, including an audiogram. *Filled* symbols indicate affected subjects

**Fig. 3:**
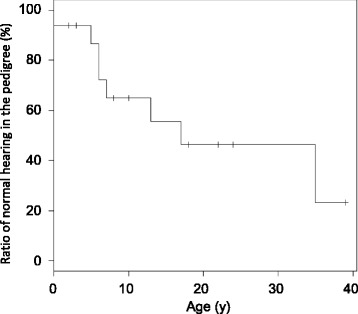
Onset age of hearing loss in family members with the m.7511T > C mutation with a maternal pattern of inheritance. Sixteen subjects in this pedigree were included in the Kaplan-Meier analysis. Note that subject I-2 was not included in the analysis due to insufficient clinical data. Vertical marks indicate censored subjects

In the family of the proband (III-5) carrying the m.7511T > C mutation, nine (I-2, II-3, II-8, III-1, III-3, III-5, IV-2, IV-3, and IV-6) of 17 maternally related members in four generations exhibited SNHL (Fig. 2). Subjects II-1, II-2, II-5, and II-7 died before they reached adulthood (≥20 y) for reasons other than illness. The phenotypes of seven family members identified as carrying the m.7511T > C mutation by genetic analysis are presented in Table 1. Five subjects, III-3, III-5, IV-2, IV-3, and IV-6, were confirmed to have SNHL by pure-tone audiometry. The onset of SNHL was congenital in one subject (IV-6), during childhood in six subjects (II-3, II-8, III-1, III-3, IV-2, and IV-3), and there was adult onset in one subject (III-5) (Table 1, Fig. 3). The severity of SNHL ranged from moderate in three subjects (III-3, III-5, and IV-3), to moderate or mild in each ear in one subject (IV-6), and mild in one subject (IV-2), whereas the other two subjects (III-7, IV-4) demonstrated normal hearing levels. The audiometric configuration was flat in one subject (IV-2), gently sloping in one ear and flat in the other ear in one subject (III-3), sloping in two subjects (III-5 and IV-3), and low-frequency SNHL was detected in one subject (IV-6). Three subjects (III-3, III-5, and IV-3) showed progression of SNHL, whereas the hearing levels of the other subjects (III-7, IV-2, IV-4, and IV-6) did not worsen. No subjects showed fluctuation of hearing. A speech discrimination test was performed for four subjects (III-3, III-5, IV-2, and IV-3) and distortion-product otoacoustic emissions (DPOAE) were tested for six subjects (III-3, III-5, III-7, IV-2, IV-3, and IV-4). The speech discrimination test indicated that all subjects had ≥ 90% discrimination, other than one, who had discrimination of 85% in the left ear. DPOAE was abnormal at all, or some, tested frequencies in all subjects. These results are consistent with hearing loss caused by inner ear disorders. In addition to SNHL, subjects III-3 and III-5 exhibited vertigo and tinnitus, whereas the other individuals carrying the m.7511T > C mutation did not present with additional symptoms.

**Table 1:** Clinical features of family members carrying the m.7511T > C mutation

Individual	Sex	Age of onset (years)	Age at time of test (years)	Severity (R/L)	Audiometric configuration (R/L)	Progression	Speech discrimination (R/L)	DPOAE
III-3	F	13	42	Moderate/Moderate	Gently sloping/Flat	Yes	95%/85%	Bilateral NR
III-5	F	35	41	Moderate/Moderate	Gently sloping/Gently sloping	Yes	90%/95%	Bilateral NR
III-7	F	–	39	Normal/Normal	NA	No	Not tested	R: NR except 1 kHz, L: NR except 1 kHz and 3 kHz
IV-2	F	7	7	Mild/Mild	Flat/Flat	No	100%/100%	NR at 4 kHz
IV-3	F	5	9	Moderate/Moderate	Steeply sloping/Gently sloping	Yes	95%/90%	Bilateral NR
IV-4	M	–	22	Normal/Normal	NA	No	Not tested	Bilateral NR
IV-6	F	0	8	Mild/Moderate	Low frequency/Low frequency	No	Not tested	Not tested

*R* right ear, *L* left ear, *NR* no response, *NA* not applicable, *DPOAE* distortion-product otoacoustic emissions

## Discussion

Among 145 probands, nine were analyzed for mtDNA gene mutations based on the presence of clinical features of mitochondrial SNHL. Five of these individuals were found to carry mutations (three cases with m.1555A > G, one with m.3243A > G, and one with m.7511T > C). These results are in agreement with previous studies reporting that m.1555A > G and m.3243A > G are the highest frequency mtDNA mutations associated with SNHL, and that other SNHL-associated mtDNA mutations occur much less frequently [4, 11]. These two most common mutations were detected in only 3% (one case of m.1555A > G and three cases of m.3243A > G) of the 136 probands who did not meet the clinical criteria. These results suggest that the clinical criteria adopted in this study are helpful for selection of candidate patients with a high probability of carrying mtDNA mutations. No known pathogenic mutations, or unknown variants, were detected in the remaining mitochondrial rRNA and tRNA genes, including *MTTK*, *MTTH*, *MTTS2*, and *MTTE*, consistent with a previous report that *MTTS1* is the third most frequent mtDNA region (after m.1555A > G in *MTRNR1* and m.3243A > G in *MTTL1*) where mutations affecting auditory function occur [12].

Recent advances in next-generation sequencing technologies have enabled the identification of multiple nuclear genes responsible for hearing loss [13–15]. However, it is technically challenging to investigate mtDNA and nuclear DNA mutations simultaneously by next-generation sequencing, since mitochondrial genes are between two and three orders of magnitude more abundant than nuclear genes in the cell [16, 17]. Therefore, pre-diagnostic selection of patients with a high probability of mitochondrial SNHL should contribute to the efficient analysis of mtDNA mutations.

At present, five *MTTS1* variants, including m.7511T > C, are confirmed as, or suspected to be, associated with nonsyndromic or syndromic SNHL [4]. *MTTS1* m.7511T > C has been identified in families with various ethnic backgrounds, including African-American, French, and Japanese (Table 2). The penetrance, severity, age of onset, and progression of SNHL across these families varies widely, making prediction of the effects of this mutation based on the clinical features of the patients difficult. The finding that patients in the family identified in this study with a homoplasmic, or highly heteroplasmic, m.7511T > C mutation had phenotypes ranging from normal hearing to moderate levels of SNHL is unique compared with previous reports, where some maternal family members with homoplasmic and heteroplasmic m.7511T > C mutations exhibited profound SNHL (Table 2). The level of hearing loss of the proband (III-3) of the family with this mutation in the present study has remained moderate over 30 years, implying that severe hearing loss is not likely to develop before the onset of presbycusis in this family.

**Table 2:** Clinical features of previously reported pedigrees with the m.7511T > C mutation

Ethnicity	Penetrance	Severity	Age of onset (years)	Audiometric configuration	Progression	Homoplasmy/heteroplasmy	Reference
African-American	36/43 (84%)	Unknown	Various	Unknown	Yes	Homo, hetero	[15]
French	7/19 (37%)	Normal to profound	3 to 33	Unknown	Yes/No^a^	Homo, hetero	[14]
French	6/19 (32%)	Normal to profound	Various	Sloping, U-shaped	No	Homo, hetero	[14]
Japanese	13/24 (54%)	Normal to profound	3 to 30	Sloping, dip, flat	Yes/No^a^	High hetero	[16]
Japanese	7/23 (30%)	Normal to profound	26 to 45	Sloping	Yes	Homo	[18]
Japanese	9/17 (53%)	Normal to moderate	0 to 40s	Sloping, flat, low-frequency	Yes/No^a^	Homo	This study

Homo, homoplasmy; hetero, heteroplasmy

^a^Progression was not always observed

Among 17 maternally related individuals with the m.7511T > C mutation in the present study, nine had SNHL. However, the penetrance in this family (53%) should be interpreted cautiously, because four subjects in generation II died before they reached adulthood. Considering the possibility that SNHL had yet to occur in subjects II-1, II-2, II-5, and II-7 at the time of their deaths, the actual penetrance of SNHL in this family is presumably higher than the calculated value. Consistent with this speculation, the penetrance in subjects in generation III and later was 6/9. Penetrance of SNHL in families with the m.7511T > C mutation in previous studies varied from approximately 30 to 84% [18–22]. Varying severity and penetrance have also been reported for m.1555A > G in *MTRNR1* and m.7445A > G in *MTTS1*, which are frequent mitochondrial mutations associated with nonsyndromic SNHL [4, 23–29]. Future studies should investigate whether other mitochondrial DNA variants and/or haplotypes have modulatory effects on penetrance or phenotype severity in families with mtDNA mutations associated with SNHL.

## Conclusion

Among the selected nine probands, five cases were determined to have pathogenic mtDNA mutations (three cases of m.1555A > G, one of m.3243A > G, and one of m.7511T > C). The m.7511T > C mutation will be difficult to predict based on the variable clinical features of the patients with this mutation. The clinical criteria used in this study are considered helpful for the efficient identification of patients likely to have mtDNA mutations associated with SNHL.

## Additional files

Additional file 1: Primers used in this study. (XLSX 11 kb)
Additional file 2: mtDNA variants detected in 5 probands fulfilling the 5 criteria. (XLSX 12 kb)
Additional file 3: Clinical features of probands carrying m.1555A > G or m.3243A > G. (XLSX 11 kb)

## Abbreviations

DPOAE: Distortion-product otoacoustic emissions
mtDNA: mitochondrial DNA
RFLP: Restriction fragment length polymorphism
